# Ser80Ile mutation and a concurrent Pro25Leu variant of the *VHL *gene in an extended Hungarian von Hippel-Lindau family

**DOI:** 10.1186/1471-2350-9-29

**Published:** 2008-04-16

**Authors:** Attila Patocs, Peter Gergics, Katalin Balogh, Miklos Toth, Ferenc Fazakas, Istvan Liko, Karoly Racz

**Affiliations:** 1 Molecular Medicine Research Group, Hungarian Academy of Sciences and Semmelweis University, Szentkirályi 46, H-1088 Budapest, Hungary; 2 2nd Department of Medicine, Faculty of Medicine, Semmelweis University, Szentkirályi 46, H-1088 Budapest, Hungary; 3 Medical and Health Science Center, University of Debrecen, H-4200, Debrecen, Hungary; 4 Richter Gedeon LTD, Budapest, Hungary

## Abstract

Von Hippel-Lindau disease (VHL) is a rare autosomal dominant disease characterized by development of cystic and tumorous lesions at multiple sites, including the brain, spinal cord, kidneys, adrenals, pancreas, epididymis and eyes. The clinical phenotype results from molecular abnormalities of the *VHL *tumor suppressor gene, mapped to human chromosome 3p25-26. The *VHL *gene encodes two functionally active VHL proteins due to the presence of two translational initiation sites separated by 53 codons. The majority of disease-causing mutations have been detected downstream of the second translational initiation site, but there are conflicting data as to whether few mutations located in the first 53 codons, such as the Pro25Leu could have a pathogenic role. In this paper we report a large Hungarian VHL type 2 family consisting of 32 members in whom a disease-causing AGT80AAT (Ser80Ile) c.239G>A, p.Ser80Ile mutation, but not the concurrent CCT25CTT (Pro25Leu) c.74C>T, p.Pro25Leu variant co-segregated with the disease. To our knowledge, the Ser80Ile mutation has not been previously described in VHL type 2 patients with high risk of pheochromocytoma and renal cell cancer. Therefore, this finding represents a novel genotype-phenotype association and VHL kindreds with Ser80Ile mutation will require careful surveillance for pheochromocytoma. We concluded that the Pro25Leu variant is a rare, neutral variant, but the presence such a rare gene variant may make genetic counseling difficult.

## Background

Von Hippel-Lindau disease (VHL) (OMIM nr. 193300) is a rare autosomal dominant multi-organ disease caused by molecular abnormalities of the *VHL *tumor suppressor gene [1]. Patients with VHL are at risk for development of retinal, cerebellar, spinal, pancreatic and renal hemangioblastomas, pulmonary and liver hemangiomas, clear-cell renal carcinomas, pheochromocytomas, endolymphatic sac tumors, multiple renal, epididymal and pancreatic cysts, cystadenomas of the epididymis and of the broad ligament, and pancreatic islet cell tumors [1-3]. Based on the presence or absence of pheochromocytoma, two main subtypes of the VHL disease have been identified. Patients with VHL type 1 are at risk to develop renal cell carcinoma and hemangioblastoma but predominantly without pheochromocytoma, while those with VHL type 2 may have all manifestations of the disease predominantly including pheochromocytoma. VHL type 2 has been subdivided into subtype 2A and 2B, with a low and high risk of renal cell carcinoma, respectively, whereas subtype 2C is a pheochromocytoma-only phenotype. The prevalence of the VHL disease varies between 1:39,000 in Germany and 1:53,000 in East Anglia [4,5]. Manifestations of the disease show variable expression and several patients may have only one manifestation [4].

The human *VHL *gene maps to chromosome 3p25-26 [1]. As predicted by Knudson's two-hit model, tumor development requires inactivation of both copies; the first hit, the germline mutation or deletion is followed by somatic alterations usually detected as loss of heterozygosity. The *VHL *gene has two translational initiation sites separated by 53 codons. Of the two proteins encoded by the *VHL *gene, the larger one contains 213 amino acids (pVHL30), whereas the shorter protein consists of 160 amino acids (pVHL18). Both proteins are functionally active and they share the same mechanism of tumor suppressor activity [6-8]. The VHL proteins form a multimeric complex with Elongin B, Elongin C, Cul2 and Rbx1. This complex is involved in ubiquitin-dependent proteolysis of large cellular proteins via controlled degradation of α-subunits of the heterodimeric transcription factor hypoxia inducible factor (HIF) [9,10]. In addition, complexes containing pVHL, Elongin B, Elongin C, Cul2 and Rbx1 target proteins are involved in cell-cycle regulation for degradation, suggesting that pVHL has a possible role in cell-cycle exit [11]. In 1999, Stebbins and co-workers presented the three-dimensional protein structure of the VHL-ElonginC-ElonginB complex. They identified two functionally active sites; the ElonginC binding site in the α-helical domain involving residues between amino acids 157 and 170, and the HIF binding site in the β-sheet domain from residue 91 to 113 [8].

The majority of VHL patients have deletions, insertions, or mutations downstream of the second translational initiation site located at codon 54 of the *VHL *gene. Interestingly, missense mutations encoding residues of the protein binding sites have been associated with VHL type 2, whereas VHL type 1 is caused either by missense or nonsense mutations affecting the hydrophobic core, or by partial gene deletions which result in a complete defect of protein function. It has been also demonstrated that some mutant pVHL, which are associated with the type 2C phenotype, may impair fibronectin matrix assembly while they retain the ability to down regulate HIF [12].

Because mutations affecting the first 53 codons of the *VHL *gene have no effect on the structure of the shorter VHL protein, it seemed particularly interesting to clarify whether these mutations could exert a pathogenic effect. In one study the Pro25Leu variant has been associated with a sporadic pheochromocytoma [13]. but other studies involving a limited number of cases failed to confirm the pathogenic role of this variant [14,15]. In this paper we report a large Hungarian VHL type 2 family with a disease-causing AGT80AAT (Ser80Ile) c.239G>A, p.Ser80Ile mutation and a concurrent CCT25CTT (Pro25Leu) c.74C>T, p.Pro25Leu variant (identification number: rs35460768 (dbSNP127) of the *VHL *gene. We show that in family members the Ser80Ile mutation, but not the Pro25Leu variant co-segregated with the disease. In order to confirm the pathogenicity of the Ser80Ile mutation and to test whether the Pro25Leu variant might be neutral, an evolutionary multiple sequence alignment analysis (MSA) combined with a Align GVGD was.performed. Three-dimensional modeling of the Ile80-mutant protein is also presented.

## Patients and methods

### Patients and data collection

**Figure 1:**
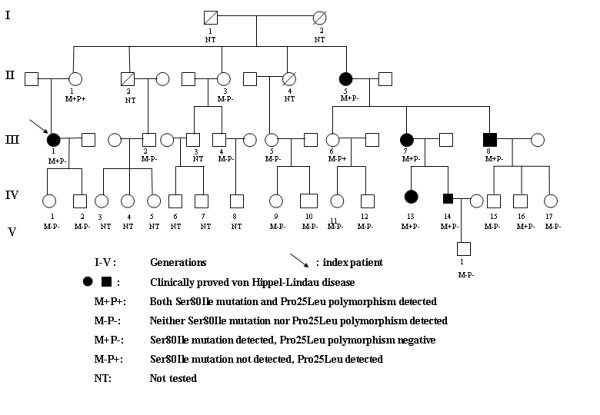
Pedigree of a Hungarian kindred with von Hippel-Lindau disease.

A large Hungarian VHL type 2 family spanning five generations and involving 32 members (Fig 1) was evaluated at the 2^nd ^Department of Medicine, Faculty of Medicine, Semmelweis University in Budapest, Hungary. Initial screening included medical history, physical examination, abdominal ultrasonography, abdominal and brain computed tomography (CT) or magnetic resonance imaging (MRI), ophthalmologic examination, routine biochemical testing and 24-h urinary catecholamine metabolite determinations.

### Screening for *VHL *gene mutations

Written informed consent was obtained from all family members who participated in the study. Genomic DNA was extracted from peripheral blood leukocytes using DNA isolation kit for mammalian blood samples (Boehringer Mannheim Corporation, Indianapolis, IN). Mutation analysis of the *VHL *gene was performed as previously described [16].

### Evolutionary alignment analysis of the VHL protein

Multiple sequence alignment (MSA) of protein sequences was performed using 3D-coffee, a freely available web-based tool for aligning multiple sequences [17]. A total of eight sequences: Mus musculus (ENSMUSG00000033933), Rattus norvegicus (ENSRNOG00000010258), Canis familiaris (ENSCAFG00000005149), Homo sapiens (ENST00000256474), Gallus gallus (ENSGALG000000013678), and evolutionary more distant species including Drosophila melanogaster (CG13221_CG13221-RA), Xenopus tropicalis (ENSXET00000001448) and Takifugu rubripes (SINFRUG00000121189) were analysed.

One popular method for measuring biochemical distances between pairs of amino acids is the Grantham Difference (18), which takes into account the composition, polarity and volume of mutant and wild-type amino acids. In this study, we have constructed a VHL protein MSA and combined a conservation score (GV) with a measure of biochemical difference between wild-type and mutant residues with respect to the alignment (GD). This extension of the Grantham Difference, called Align GVGD, has previously been successfully applied to *BRCA1 *and *TP53 *genes (19, 20). Align GVGD is freely available online (21).

### Three-dimensional structure modeling of the VHL protein

The coordinates of the pVHL were obtained from 1vcb_a.pdb structure file [8]. The three-dimensional image was generated with Swiss-PdbViewer in combination with POV-Ray [22].

## Results

### Genotype-phenotype associations

The index patient was operated for pheochromocytoma and renal cell carcinoma diagnosed at the age of 34 yr and 40 yr, respectively. She also developed a brain hemangioblastoma at the age of 42 yr. Genetic screening indicated that she had a heterozygous AGT80AAT (Ser80Ile) c.239G>A, p.Ser80Ile mutation of the *VHL *gene. The 70-yr-old mother of the index patient proved to be not only a gene carrier for the Ser80Ile mutation, but she also had the CCT25CTT (Pro25Leu) c.74C>T, p.Pro25Leu variant (identification number: rs35460768 (dbSNP127)), both in heterozygous forms. However, the mother showed no clinical, biochemical or radiological evidence of VHL-associated tumors. The absence of the Pro25Leu variant in the index patient indicated, that the Ser80Ile mutation and the Pro25Leu variant in the mother were present in separate alleles.

In addition to the index patient and her mother, the Ser80Ile mutation was detected in other 5 members of the family (III/7, III/8, IV/13, IV/14 and IV/16) (Fig 1), of which all but one member (III/7, III/8, IV/13 and IV/14) had clinical manifestations of the disease.

Importantly, pheochromocytomas were identified in family members III/8 and IV/14, of whom, in patient IV/14 this was the first sign of the disease manifested at the age of 10 years and involved both adrenals. Other typical manifestations included brain hemangioblastomas in family members: III/7, III/8 and IV/13, retina hemangioblastomas in family members III/7, III/8, IV/13 and IV/14, and renal cysts in family member IV/13. In addition, family history revealed that one sister of the mother of the index patient (II/5) died due to bilateral renal cell carcinomas. Another sister of the mother of the index patient (II.4) died because of a metastatic pancreatic tumor without histological confirmation and, therefore, this family member was not considered as having VHL. The age, clinical manifestations, and the age at diagnosis of VHL-associated lesions in carriers of the Ser80Ile mutation and in those with clinically proven VHL-associated tumors are summarized in Table 1.

**Table 1:** Clinical manifestations of VHL disease in carriers of the Ser80Ile mutation and/or the Pro25Leu variant and in those with clinically proven VHL-associated tumors

**Family member (age, years)**	**Renal cell carcinoma (age at presentation, years)**	**Brain heman-gioblastoma (age at presentation, years)**	**Pheochromo-cytoma (age at presentation, years)**	**Retina heman-gioblastoma (age at presentation, years)**	**Other clinical manifestations**	**Result of genetic screening**
II/1 (70)	-	-	-	-	-	Pro25Leu Ser80Ile
III/6 (44)	-	-	-	-	-	Pro25Leu
II/5 (63)	+ bilateral (45)	-	-	-		ND
III/1* (42)	+ bilateral (40)	+ (42)	+ (34)	-	-	Ser80Ile
III/7 (47)	-	+ (37)	-	+ (18)	-	Ser80Ile
III/8 (39)	-	+ (38)	+ (39)	+ (29)	-	Ser80Ile
IV/13 (27)	-	+ (22)	-	+ (23)	renal cysts (20)	Ser80Ile
IV/14 (26)	-	-	+ bilateral (10)	+ (26)	-	Ser80Ile
IV/16 (16)	-	-	-	-	-	Ser80Ile

*, index patient; -, present; +, absent; ND, not determined (DNA for genetic testing was not available)

In addition to the mother of the index patient, the Pro25Leu variant was detected on another family member (III/6) (Fig 1), who was clinically healthy.

The prevalence of the Pro25Leu variant was tested in 16 members of other 8 VHL families and in 39 patients with sporadic pheochromocytomas, but none of the patients had this variant.

### Evolutionary alignment analysis of the *VHL *gene

**Figure 2:**
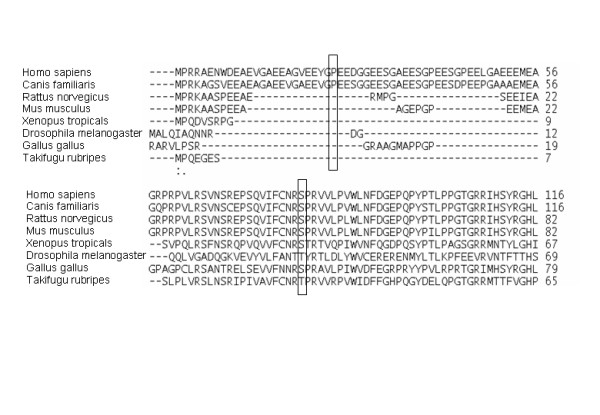
Alignment analysis of the VHL protein from Mus musculus (ENSMUSG00000033933), Rattus norvegicus (ENSRNOG00000010258), Canis familiaris (ENSCAFG00000005149), Homo sapiens (ENST00000256474), Gallus gallus (ENSGALG000000013678), Drosophila melanogaster (CG13221_CG13221-RA), Xenopus tropicalis (ENSXET00000001448) and Takifugu rubripes (SINFRUG00000121189).

As shown in Fig. 2., the Ser at amino acid position 80 represents a highly conserved amino acid residue among different species, including rat, mouse, dog, chicken and an evolutionary more distant species, Xenopus tropicalis. By contrast, the Pro at amino acid position 25 failed to show a conserved pattern. Using Align-GVGD criteria both variants were predicted as neutral fulfilling the criteria 0<GV ≤ 61,3 and GD>0 (the calculated GV and GD scores for Pro25Leu polymorphism were 218.82 and 4.86 and for Ser80Ile 219.56 and 3.24, respectively).

### Effect of change of Ser80 to Ile80 on protein structure

**Figure 3:**
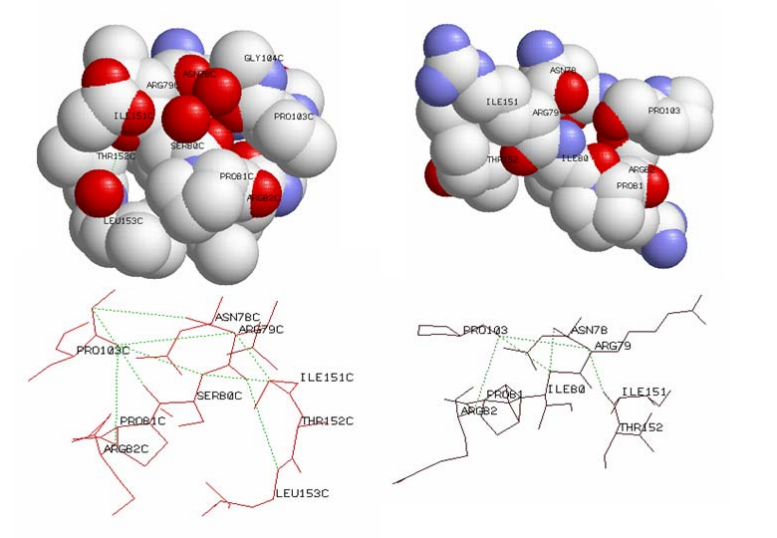
**Three-dimensional computational modeling of the Ser80 and Ile80 residues using CPK diagram (upper part) and schematic representation of hydrogen-bonds between residues Gly104 and Asn78, between Arg79 and Ile151, between Ser80 and Ile151, beween Ser80 and Pro103, between Ser80 and Leu153, and between Pro103 and Arg82 in wild type pVHL and the consequences of the Ser to Ile change at amino acid position 80 (lower part).** All figures were generated with Swiss-PdbViewer in combination with POV-Ray.

The three-dimensional model of the pVHL is available from residue Met54, which allowed us to analyse functional consequences of the Ser80Ile mutation using a three-dimensional computational modeling. The CPK diagram in Fig. 3 illustrates both the wild-type Ser80 and the mutant Ile80 proteins. The Ser80 residue makes tight junctions with Pro103 and Ile151 by hydrogen-bonds between the amino group of Ser80 and the carboxyl oxygen of Pro103 and Ile151. The Ser80 residue is located on the same side where ElonginC binds, and residues Pro81 and Arg82 are involved in direct binding. The Asn78 residue from the β-domain, together with hydrophobic core residues (Pro86, Phe76, Phe119, Trp117 and Val130) have important roles for structural integrity of the β-sandwich [8]. A change of Ser to Ile at position 80 disturbs this integrity of the β-sandwich due to a change in the orientation of the Arg82 residue, which disturbs its linker role and, consequently, the hydrogen bond between Arg82 and Leu153 disappears. It has been already demonstrated that the Arg82 residue plays a central role in structural integrity of the pVHL, as it makes significant hydrogen-bond contacts with Leu153, Val155, Lys159 and Arg161 and, through these contacts; it is involved in interactions between pVHL and ElonginC [8].

## Discussion

In this paper we describe a large Hungarian VHL type 2 family consisting of 32 members, in whom a disease-causing AGT80AAT (Ser80Ile) c.239G>A, p.Ser80Ile mutation and a concurrent CCT25CTT (Pro25Leu) c.74C>T, p.Pro25Leu variant were identified. Screening of family members indicated that 7 family members had the Ser80Ile mutation and 2 members had the Pro25Leu variant, and that the two genetic alterations were transmitted in separate alleles. More importantly, we showed that the Ser80Ile mutation, but not the Pro25Leu variant co-segregated with the VHL type 2 phenotype and that the Pro25Leu variant never occurred in family members who had manifestations of the disease. However, one family member who had both genetic alterations and one member with the Ser80Ile mutation without the Pro25Leu variant showed no manifestations of the disease, suggesting that this variant is not pathogenic for VHL.

In order to predict the pathological role of the Pro25Leu and Ser80Ile variants, first we constructed a multiple sequence alignment using evolutionary distant species. It has been shown previously that the addition of more distantly related sequences is essential for a more accurate prediction (20). However, in our case this approach revealed that both variants might be neutral, which is in contrast with earlier clinical findings suggesting the disease causing role of the Ser80Ile but not of the Pro25Leu. The Ser80Ile has been previously reported only in one VHL patient without clear cell renal carcinoma [23], but several other missense mutations at codon 80 have been described. The Ser80Gly mutation has been detected in a 12-yr-old patient with bilateral pheochromocytomas and multiple congenital malformations [24] and in a patient with pheochromocytoma [25]. The Ser80Asn mutation was identified in a Slovakian family with VHL type 1 phenotype including pancreatic islet tumor in one affected individual [26]. In addition, the pathogenic role of Ser80Arg [27,28] and Ser80Asn mutations has been also documented in VHL patients [25]. Our study shows for the first time that the Ser80Ile mutation is associated with bilateral pheochromocytoma presented as a first manifestation of the disease, which points out that VHL patients harboring this mutation require careful screening for pheochromocytoma.

The association between various missense mutations at codon 80 of the *VHL *gene and VHL disease in previous studies and ours is not unexpected, since our evolutionary alignment analysis showed that in this position the Ser is a highly conserved amino acid residue among different species. Moreover, computational protein modeling of a change of Ser to Ile at position 80 indicated potentially important consequences on three-dimensional structure of the pVHL. Ser80 is a polar amino acid located in the hydrophobic core of the β-domain close to the α-β interface of pVHL and ElonginC interaction. According to our protein modeling, the change of Ser to a larger and nonpolar Ile at amino acid position 80 results in a complex rearrangement of the three-dimensional structure of the protein binding interface, which disturbs HIF1α and fibronectin binding [29]. Miller and co-workers have already demonstrated that *VHL *gene mutations within L1 and L7 loops distrupt the dynamic coupling of the pVHL and HIF-1α and that this may be an important mechanism for the development of tumors [30]. Other studies using three-dimensional modeling and a computer algorithm FOLDEF provided a quantitative estimation of the importance of the interactions contributing to the stability of proteins and protein complexes [31]. In the case of the Ser80Ile, the free energy difference between the wild type and mutant proteins was 12.2 kcal/mol, which is higher than the previously established cut-off value for the development of clear-cell renal carcinoma or pheochromocytoma [32].

Although mutations affecting the first 53 codons of the *VHL *gene have no effect on the structure of the shorter VHL protein, a few mutations at codons 25, 38, 46 and 52 have been considered to exert a pathogenic role. The Pro25Leu and Ser38Pro have been identified in patients with pheochromocytomas, whereas the Glu46Stop and Glu52Lys have been associated with VHL disease [32,27]. However, Rothberg et al. reported a VHL patient with two *VHL *gene mutations, Pro25Leu and Pro86Arg, in whom the Pro86Arg was considered more likely to be pathogenic than the Pro25Leu based on an allelic frequency of 0.5% of the latter variant in anonymized DNA samples [14]. This Pro25Leu variant was detected in other healthy VHL family members from Poland and North America [33,14]. In agreement with these observations, the Pro25Leu carrier in our family had no manifestations of the disease, suggesting that this variant does not represent a disease-causing mutation.

## Conclusion

The Ser80Ile mutation of the *vhl *gene in a large Hungarian kindred was found to be associated with VHL type 2 presenting with both pheochromocytoma and renal cell cancer. Therefore, patients with this mutation require careful surveillance, including search for pheochromocytoma. The Pro25Leu variant in this family apeared to be neutral, but its co-existence made genetic counseling difficult.

## Abbreviations

VHL: von Hippel-lindau disease; pVHL: von Hippel-Lindau protein; HIF: hypoxia inducible factor.

## Competing interests

The author(s) declare that they have no competing interests.

## Authors' contributions

Acquisition of data: AP, KB, PG, FF performed the molecular biological analysis, AP, FF and IL participated in alignment analysis and in three dimensional protein modeling, MT and KR obtained the clinical data. Analysis and interpretation of data: AP, FF, IL, MT and KR. Drafting of the manuscript: AP, KB, PG, MT, IL and KR. Critical revision of the manuscript for important intellectual content: AP, IL, MT and KR. Administrative, technical, or material support: AP, PG, KB, FF, IL, MT and KR. Study supervision: KR.

## Pre-publication history

The pre-publication history for this paper can be accessed here:


